# Blood pressure control as an intervention to prevent dementia

**DOI:** 10.1016/S1474-4422(19)30288-1

**Published:** 2019-08-20

**Authors:** Lenore J Launer

**Affiliations:** Intramural Research Program, National Institute on Aging, National Institutes of Health, Bethesda, Baltimore, MD 21224, USA

Observational findings of a relationship between blood pressure during midlife and cognitive brain outcomes have been replicated in different populations, but negative findings have also been reported.^1^ Often based on cross-sectional studies or on data from very old people (ie, >80 years of age), interpretations of these negative studies have not accounted for issues such as reverse causation.^2^ As a consequence of inconsistencies in the published literature, blood pressure lowering has not been accepted as a candidate for intervention against cognitive impairment or dementia—a trial was needed.

The SPRINT MIND trial detected a cognitive benefit of blood pressure lowering in a large cohort of people aged 50 years and older with diverse ethnic origins.^3^ This trial strengthens the observational study conclusions that blood pressure is involved in modulating risk for dementia and underlines the importance of linking findings in risk factors during middle age with outcomes in older people.

In *The Lancet Neurology*, Lane and colleagues^4^ bring new insights into blood pressure-cognitive-related outcomes, drawing from data collected on a subsample of the 1946 British birth cohort, in a design that controls for reverse causation. With data on multiple measures done at around 8-year intervals between the ages of 36 years and 70 years, the authors examined the associations of levels and changes in blood pressure with brain structure and function and with amyloid load, a biomarker for Alzheimer’s disease pathology. The study addresses windows of opportunity for prevention of dementia and provides data testing the hypothesis that vascular damage contributes to Alzheimer’s disease pathology.

This study showed that the window of opportunity spans middle age, peaking in the 40s. During this period, both heightened and increases in systolic and diastolic blood pressure were associated with more white matter lesions, an indicator of vascular damage. Higher diastolic blood pressure and change in diastolic blood pressure in the period of 36–43 years of age were associated with smaller whole-brain volume later in life. These results were robust to correction for other cardiovascular risk factors at 70 years of age.

However, blood pressure was not associated with cognition but, as the authors note, there might not have been enough variability to detect differences in cognition. Absence of association might also reflect life course differences. The authors adjusted their findings for early life cognitive performance, a factor that has been shown previously to be significantly correlated with later life cognitive performance in this cohort;^5^ at older ages, changes in cognition might lag behind changes in brain structure.

This new study finds no association between high blood pressure and amyloid pathology measured on PET. As the authors note, this negative finding suggests that high blood pressure more likely affects dementia through small vessel disease than through amyloid-related pathways. However, a negative finding does not necessarily provide evidence against a vascular contribution to Alzheimer’s disease pathology. For instance, PET amyloid imaging cannot identify precursors to amyloid deposition or be used to investigate the interaction between neurodegenerative and vascular processes on a micro level.

The findings from this study motivate a deeper dive into the role of blood pressure components and brain health. For example, most studies have examined the level of blood pressure in relation to brain outcomes, but other components of blood pressure might be physiologically important, such as pulse wave velocity, or other manifestations of hypertension, such as isolated high systolic or diastolic blood pressure. Additionally, besides macro structure changes in the brain, data are scarce, particularly from cohort studies, on the contribution of elevated blood pressure to cerebral microstructural damage and cerebral blood flow and metabolism, or to disturbances in the blood brain barrier, autoregulation, and clearance of toxic substances from the brain. Whether there are phases and dependencies in which these brain pathologies emerge that are differentially related to vascular and haemodynamic changes is also unknown. Several clinical questions also remain. For example, should treatment options be the same for cardiovascular disease or are specific regimes or drugs more effective than others in reducing risk of cognitive impairment; and should different protocols be developed depending on age, comorbidity, and ethnicity, as cardiovascular guidelines propose?

Although there are several major translational efforts to more completely understand the complexity of blood pressure-cognitive-related outcomes, the association between blood pressure and vascular brain pathology is unlikely to be a chance finding. Millions of individuals have unhealthy blood pressure. Immediate attention should be given to efforts to control blood pressure through clinical services and public health interventions, and to alleviate the barriers to delivery and uptake of these public-health messages.^6^
